# Substitution of hospital care with Primary Care Plus: differences in referral patterns according to specialty, specialist and diagnosis group

**DOI:** 10.1186/s12875-019-0961-4

**Published:** 2019-06-11

**Authors:** Paul Smeele, Mariëlle E. A. L. Kroese, Marieke D. Spreeuwenberg, Dirk Ruwaard

**Affiliations:** 1 Department of Health Services Research, Care and Public Health Research Institute, Faculty of Health Medicine and Life Sciences, Maastricht University, PO Box 616, 6200 MD Maastricht, The Netherlands; 2 Research Centre for Technology in Care, Zuyd University of Applied Sciences, Heerlen, The Netherlands

**Keywords:** Primary care, Primary Care Plus, Substitution, Integrated care, Collaboration, Referrals

## Abstract

**Background:**

Primary Care Plus (PC+) is an intervention where patients consult specialists in a primary care setting outside the hospital. Two facilities have been founded in the city of Maastricht, the Netherlands. Main aim is to achieve substitution of hospital care with primary care and hence reduce costs. The objective of this study is to evaluate referral patterns per specialty, specialist and diagnosis group, as input for deliberations to optimise substitution.

**Methods:**

Prospectively collected referral data after PC+ consultations between November 2014 and March 2016 was analysed for eight participating specialties. Primary outcomes were differences in referral patterns per specialty, specialist and diagnosis group. Absolute counts and percentages were recorded for categorical variables, means and standard deviations for continuous variables. Statistical analyses were performed using IBM SPSS Statistics 23 (SPSS Inc., Chicago, IL).

**Results:**

In total 4536 patients were seen in PC+; 3132 (69.0%) were referred back to the general practitioner (GP), whereas 1275 (28.1%) were referred to secondary care. Referral information of 130 (2.9%) patients was unknown. Large differences in referral numbers to secondary care after PC+ consultation were found between specialties (from 8.6% (gynaecology) to 43.8% (orthopaedic surgery)), specialists (14.5 to 65.2%) and diagnosis groups (11.1 to 93.4%).

**Conclusions:**

Wide variation in referral numbers to secondary care between specialties, specialists and diagnosis groups exists after PC+ consultations. This data indicates that deliberation and further research is needed in order to optimize substitution initiatives like PC+.

## Background

Growing technological possibilities, ageing populations and the resulting rising number of chronically ill are putting a steadily increasing burden on health systems in OECD countries, which spend on average 8.9% of their Gross Domestic Product (GDP) on healthcare [1]. Despite the post-crisis slowdown in health spending growth, concerns about the financial sustainability of health systems of OECD countries remain high.

In the Netherlands, which ranks second (together with Switzerland) on the list of largest per capita spenders on healthcare (after the United States), the share of GDP allocated to healthcare spending in 2013 was 11.1% [2]. Without structural cost-containing efforts, this number is expected to rise to 22–31% in 2040, threatening future accessibility, affordability and hence, sustainability of Dutch healthcare [3].

To slow down healthcare costs in these countries while improving quality of care, innovations in healthcare should simultaneously strive to accomplish Berwick’s Triple Aim: (1) improve the health of populations, (2) improve patients’ experiences of care and (3) reduce the per capita costs of care [4]. Substitution of costly hospital care with more affordable primary care is seen as a promising solution that has the potential to do so [3, 5].

To improve the quality and financial sustainability of healthcare in the Netherlands, in 2013 nine pioneer sites were appointed to experiment with innovative health care solutions [6]. In one of those pioneer sites (Blue Care in Maastricht-Heuvelland), an intervention called Primary Care Plus (PC+) was developed. PC+ facilitates specialist consultations outside the hospital in a primary care setting and explores the effectiveness of substitution of hospital care with primary care. The initiative is geared towards accomplishing the Triple Aim by reducing unnecessary referrals to secondary care and reinforcing the gatekeeper role of the GP.

At present, few studies exploring the effectiveness of substitution initiatives have been published [7–13], most of which examined small-scale substitution initiatives with shared GP-specialist consultations in GP practices. All of the studies showed reduced referrals to secondary care, indicating that substitution was taking place. Among those studies, a pilot study into PC+ in 2013 explored the feasibility of specialist consultations by five specialties (orthopaedic surgery, dermatology, neurology, cardiology and internal medicine) in ten GP practices across Maastricht-Heuvelland, The Netherlands. The study provided strong indications that PC+ has the potential to achieve substitution of care, with high patient and stakeholder satisfaction [7, 8]. However, PC+ in this form was not continued due to efficiency constraints.

Based on the experiences of the pilot study, in 2014 two facilities were established in which specialists provide consultations outside the hospital in a primary care setting. Over time, an increasing number of specialties have participated. Nevertheless, it remains unclear whether these specialties and specialists are suitable to participate in substitution initiatives like PC+.

Therefore, this study describes referral patterns after PC+ consultations (back to the GP and to secondary care) for eight specialties involved in PC+, and explores differences in referral patterns between the specialties and between specialists and diagnosis groups. This knowledge is vital in deciding which specialties are suitable, what part of secondary care is appropriate for substitution with primary care, and whether initiatives like PC+ are feasible solutions that potentially contribute to the sustainability of OECD health systems.

## Methods

### Study setting

The PC+ intervention was developed within pioneer site Blue Care in Maastricht-Heuvelland. Blue Care was established as a partnership between the primary care organisation Care in Development (In Dutch ‘Zorg In Ontwikkeling’ (ZIO)), the Maastricht University Medical Centre (MUMC+), the patient representative foundation House of Care, and the health insurer VGZ. Since November 2014, PC+ consists of two facilities outside the hospital in the Eastern and Western parts of the city of Maastricht. At these facilities, medical specialists perform shortened consultations (maximum of two consultations per patient) for low-complex care requests in a primary care setting with primary care financing. PC+ consultation rooms are equipped with a minimum of diagnostic tools (comparable to the primary care setting). In total 81 GPs from 55 practices (affiliated with ZIO) were able to refer patients to PC+ consultations when they were unsure about a diagnosis, treatment or the need to refer a patient to hospital care. After consultation, the specialist provides written feedback to the referring GP in which either tailored treatment advice or a recommendation to refer the patient to secondary care is outlined. The specialist is able to deliberate with the GP, but the GP remains responsible for the patient throughout the whole care trajectory in PC+.

### Study design and population

An analysis of prospectively gathered data was conducted. Information on whether a patient was sent back to the GP or referred to secondary care after a PC+ consultation was retrieved from the PC+ databases. All patients that were seen by the specialists of eight participating specialties (internal medicine (INT), neurology (NEU), orthopaedic surgery (ORT), dermatology (DER), ophthalmology (OPT), rheumatology (REU), otorhinolaryngology (ENT), and gynaecology (GYN)) in one of the two facilities between November 2014 and March 2016 were included. Agreements were made between stakeholders about patients considered eligible for PC+ consultations. Inclusion criteria for PC+ were: all patients with non-acute complaints in need of low-complexity care that on first sight did not necessarily require hospital facilities. Exclusion criteria were: (1) patients with acute health problems that required immediate referral, (2) complex health problems that required more extensive diagnostics or treatments not available in the PC+ setting, and (3) complaints related to a prior diagnosis of diabetes mellitus, chronic obstructive pulmonary disease and/or vascular risks, i.e. conditions for which bundled payment contracts exist between the health insurers and ZIO.

### Database

The PC+ database contained the following information for all patients that were seen in PC+ between November 2014 and March 2016: date of consultation, age and gender, number of consultations, consulted specialty, consulted specialist and a diagnosis-code, the DTC-code. DTC stands for Diagnosis-Treatment-Combination and comprises several diagnoses that use comparable resources and that are reimbursed equally [8]. The specialist registered the DTC-code after each consultation.

### Statistical analysis

Data were analysed using absolute counts and percentages for categorical variables; means and standard deviations were used for continuous variables. Patient characteristics (age and gender) were described and compared between the eight specialties using ANOVA with Bonferroni post hoc tests and Chi-square tests, respectively.

The validity of the referral information in the PC+ databases was tested by taking random samples of 10% of the patients of each specialty (with a minimum of 20 patients per sample) from the PC+ databases. For each patient in these random samples, the referral outcome (referral to secondary care or back to the GP) was compared with true hospital visits according to the MUMC+ hospital database. Differences in mean age and gender distribution between the random sample and the entire group were analysed by means of Z-tests and Chi-square tests, respectively.

The number of consultations per patient in PC+ was described for all specialties, and mean differences between specialities were analysed using ANOVA with Bonferroni Post Hoc tests.

For the speciality that referred most patients to secondary care, referral numbers were cross tabulated against the specialist-code and the DTC-codes in order to reveal differences in referral numbers between specialists and between certain diagnosis groups. This was also done for a speciality with a similar group size that referred considerably fewer patients to secondary care. Diagnosis groups were based on a Diagnosis-Treatment-Combination (DTC)-code that was attributed to a patient after consultation in PC+.

The normality of distribution of continuous variables was checked by analysing skewness and kurtosis. If variables were not normally distributed, non-parametric tests were used. *P-*values smaller than 0.05 were considered statistically significant. Analyses were performed using IBM SPSS Statistics 23 (SPSS Inc., Chicago, IL).

### Medical research and ethics committee review

The study was approved by the Medical Research and Ethics Committee of the Maastricht University Medical Centre, Maastricht, the Netherlands in 2014 (number:14–4-136).

## Results

### Referral patterns and validation according to specialty

Between November 2014 and March 2016 4536 patients were seen in PC+. Table 1 shows the number of patients per specialty and the distribution of their population characteristics, age, and gender. The mean age was 52.7 years (SD = 19.6), with a minimum of 35.8 years (SD = 16.7) for GYN and a maximum of 58.7 years (SD = 18.7) for OPT. GYN patients were younger than patients of all other specialties (all *p* < 0.001). DER patients were younger than patients of all other specialties but GYN (*p* < 0.001 – *p* = 0.016). OPT patients were older than patients from NEU, DER, ORT, REU and GYN (*p* = 0.049 – *p* < 0.001). More females than males were seen in PC+ (2714 (59.8%), *p* < 0.001)). GYN (as expected) and REU treated more female patients in PC+ than the other specialities (*p* < 0.001 – *p* = 0.009). Furthermore, internists treated more female patients than ORT (*p* = 0.046) and ENT (*p* = 0.002).

**Table 1:** Population characteristics of patients seen at PC+ from November 2014 to March 2016

	Total	INT	NEU	DER	ORT	OPT	ENT	REU	GYN
Patients, n (%)	4536 (100.0)	162 (3.6)	276 (6.1)	1472 (32.5)	820 (18.1)	570 (12.6)	872 (19.2)	306 (6.7)	58 (1.3)
Age (y), mean ± SD	52.7 ± 19.6	53.9 ± 19.7	54.3 ± 17.3	48.4 ± 21.1	53.0 ± 17.6	58.7 ± 18.7	55.9 ± 19.6	53.7 ± 14.3	35.7 ± 16.7
p-value	<0.001								
Gender									
Female, n (%)	2714 (59.8)	114 (70.4)	169 (61.2)	877 (59.6)	471 (57.4)	328 (57.5)	468 (53.7)	229 (74.8)	58 (100.0)
Male, n (%)	1822 (40.2)	48 (29.6)	107 (38.8)	595 (40.4)	349 (42.6)	242 (42.5)	404 (46.3)	77 (25.2)	0 (0.0)

The mean number of consultations per case in PC+ was 1.14 (SD = 0.37) with a minimum of 1.02 (SD = 0.16) for OPT and a maximum of 1.37 (SD = 0.50) for REU (Table 2). Most patients (*n* = 3915; 86.3%) consulted PC+ once, 595 patients (13.1%) twice and 26 (0.6%) three times.

**Table 2:** Consultations in PC+ per case per specialty

	Mean ± SD	*P*-value
Group (*n* = 4536)	1.14 ± 0.37	< 0.001
OPT (*n* = 570)	1.02 ± 0.16	
INT (*n* = 162)	1.09 ± 0.30	
ORT (*n* = 820)	1.09 ± 0.29	
NEU (*n* = 276)	1.12 ± 0.32	
ENT (*n* = 872)	1.13 ± 0.34	
GYN (*n* = 58)	1.14 ± 0.35	
DER (*n* = 1472)	1.19 ± 0.43	
REU (*n* = 306)	1.37 ± 0.50	

Table 3 presents the outcomes of PC+ consultations as registered in the PC+ databases (Total group) and of the random samples taken from the hospital data (Random samples: 10% of cases from each specialty, with a minimum of 20 patients per sample). As presented in the left column of the table, 28.1% (*n* = 1275) of patients were referred to secondary care after consultation in PC+, whereas 69.0% (*n* = 3132) were referred back to the GP. On checking the validity of referral numbers retrieved from the PC+ databases (by random sampling), a small discrepancy was found between these numbers and true referral information based on the hospital database (34.3% to secondary care and 65.7% back to the GP).

**Table 3:** Outcome of PC+ consultation for the total group and the random sample

	Total group (*n* = 4536)				Random samples (*n* = 472)				*P*-value
Mean age ± SD	52.7		±19.6		51.8		±18.8		0.314
Gender	Female	2714	(59.8%)		297		(62.9%)		
	Male	1822	(40.2%)		175		(37.1%)		0.368
	Back to GP		Referral to SC		Back to GP		Referral to SC		
	n	%	n	%	n	%	n	%	
Total*	3132	(69.0)	1275	(28.1)	310	(65.7)	162	(34.3)	
GYN	53	(91.4)	5	(8.6)	18	(90.0)	2	(10.0)	
INT	136	(84.0)	26	(16.0)	17	(85.0)	3	(15.0)	
ENT	687	(78.8)	180	(20.6)	67	(77.0)	20	(23.0)	
DER	1083	(73.6)	305	(20.7)	100	(68.0)	46	(31.3)	
OPT	365	(64.0)	202	(35.4)	37	(64.9)	21	(35.1)	
NEU	174	(63.0)	91	(33.0)	17	(60.7)	11	(39.3)	
REU	182	(59.5)	107	(35.0)	16	(51.6)	15	(48.4)	
ORT	451	(55.0)	359	(43.8)	38	(46.3)	44	(53.7)	

*Total represents all patients that were seen in PC+ of which referral information after consultation was known. For 2.9% the outcome after PC+ consultation was unknown, which explains why percentages in the left column do not add up to 100%

The random sample consisted of 472 (10.4%) patients, with a mean age of 51.8 years (SD = 18.8) and a gender distribution of 297 (62.9%) females and 175 (37.1%) males. No significant differences in mean age (0.9 years, Z-test: *p* = 0.314) and gender distribution (*p* = 0.368) were found compared with the total group. No significant differences in patient characteristics were found between the random sample and the PC+ database.

### Referral patterns according to specialist and diagnosis group

As described in the methods section, analyses of referral patterns between specialists and between diagnosis groups were conducted for the specialties that referred most patients (43.8%) to secondary care (ORT, *n* = 820), as well as for a specialty with a comparable group size that referred considerably fewer patients (20.6%) to secondary care (ENT, *n* = 872).

Table 4 presents referral numbers per orthopaedic surgeon (O1 – O12) and per ENT specialist (E1 – E4). Specialists who had seen fewer than 20 cases in PC+ were left out of the analysis. A wider variation in referrals to secondary care between orthopaedic surgeons (range = 46.0% (min = 19.2% – max = 65.2%)) than between ENT-specialists (range = 12.3% (min = 14.5% – max = 26.8%)) was found.

**Table 4:** Outcome of PC+ consultation per specialist

	Back to GP		Referral to SC		Unknown		Total	
	n	(%)	n	(%)	n	(%)	n	(%)
Orthopaedic surgeon								
O1	21	(80.8)	5	(19.2)	0	(0.0)	26	(100.0)
O2	95	(64.6)	50	(34.0)	2	(1.4)	147	(100.0)
O3	91	(63.2)	53	(36.8)	0	(0.0)	144	(100.0)
O4	42	(60.9)	26	(37.7)	1	(1.4)	69	(100.0)
O5	70	(53.0)	57	(43.2)	5	(3.8)	132	(100.0)
O6	13	(52.0)	12	(48.0)	0	(0.0)	25	(100.0)
O7	24	(49.0)	25	(51.0)	0	(0.0)	49	(100.0)
O8	11	(47.8)	11	(47.8)	1	(4.3)	23	(100.0)
O9	32	(47.8)	34	(50.7)	1	(1.5)	67	(100.0)
O10	9	(39.1)	14	(60.9)	0	(0.0)	23	(100.0)
O11	22	(34.9)	41	(65.1)	0	(0.0)	63	(100.0)
O12	8	(34.8)	15	(65.2)	0	(0.0)	23	(100.0)
ENT Specialist								
E1	265	(85.5)	45	(14.5)	0	(0.0)	310	(100.0)
E2	101	(77.1)	30	(22.9)	0	(0.0)	131	(100.0)
E3	256	(76.0)	78	(23.1)	3	(0.9)	337	(100.0)
E4	59	(72.0)	22	(26.8)	1	(1.2)	82	(100.0)

Table 5 provides an overview of the most common diagnosis groups (based on DTC-codes) of ORT and ENT. Diagnosis groups with fewer than 5 cases were left out of the analysis. For ORT, referrals to secondary care ranged from 11.1% for chronic bursitis of the hip to 93.4% for meniscus injuries. For ENT, referrals to secondary care ranged from 0.0% in case no ENT disease was found to 66.7% for osseous anomalies.

**Table 5:** Outcome of PC+ consultation per diagnosis-group (ORT and ENT)

	Back to GP		Referral to SC		Unknown		Total	
	n	(%)	n	(%)	n	(%)	n	(%)
Diagnosis group ORT								
Chronic bursitis hip	16	(89.9)	2	(11.1)	0	(0.0)	18	(100.0)
Hallux rigidus	10	(83.3)	2	(16.7)	0	(0.0)	12	(100.0)
Patellofemoral Pain Syndrome (PFPS)	29	(80.6)	6	(16.7)	1	(2.8)	36	(100.0)
Arthrosis foot/ankle	10	(76.9)	3	(23.1)	0	(0.0)	13	(100.0)
Fractures/trauma	14	(66.7)	7	(33.3)	0	(0.0)	21	(100.0)
Non-specific low back pain	11	(64.7)	5	(29.4)	1	(5.9)	17	(100.0)
Arthrosis knee	60	(64.5)	33	(35.5)	0	(0.0)	93	(100.0)
Other complaints foot/ankle	36	(64.3)	20	(35.7)	0	(0.0)	62	(100.0)
Tendinitis s.s./biceps/impingement	40	(62.5)	21	(32.8)	3	(4.7)	64	(100.0)
Tendinitis	17	(58.6)	12	(41.4)	0	(0.0)	29	(100.0)
Arthrosis hand/wrist	14	(58.3)	9	(37.5)	1	(4.2)	24	(100.0)
Epicondylitis med./lat.	8	(57.1)	5	(35.7)	1	(7.1)	14	(100.0)
Hallux valgus	6	(54.5)	5	(45.5)	0	(0.0)	11	(100.0)
Arthrosis/spondylosis spinal cord	15	(50.0)	14	(46.7)	1	(3.3)	30	(100.0)
Rupture rotator cuff/biceps	11	(45.8)	13	(54.2)	0	(0.0)	24	(100.0)
Arthrosis hip	15	(35.7)	27	(64.3)	0	(0.0)	42	(100.0)
Meniscus injury	5	(6.6)	71	(93.4)	0	(0.0)	76	(100.0)
Diagnosis group ENT								
No ENT disease found	17	(100.0)	0	(0.0)	0	(0.0)	17	(100.0)
Globus/swallowing complaints	109	(88.6)	14	(11.4)	0	(0.0)	123	(100.0)
Allergy/hyper reactivity	46	(88.5)	6	(11.5)	0	(0.0)	52	(100.0)
Cerumen, otitis externa, corpus alienum	94	(85.5)	15	(13.6)	1	(0.9)	110	(100.0)
Perceptive deafness	171	(84.7)	31	(15.3)	0	(0.0)	202	(100.0)
Epistaxis	21	(84.0)	3	(12.0)	1	(4.0)	25	(100.0)
Dysphonia	58	(80.6)	14	(19.4)	0	(0.0)	72	(100.0)
ENT complaints other	12	(80.0)	2	(13.3)	1	(6.7)	15	(100.0)
Naso-septal anomalies	17	(68.0)	8	(32.0)	0	(0.0)	25	(100.0)
Chronic otitis media	10	(66.7)	5	(33.3)	0	(0.0)	15	(100.0)
OMA, OME, tuba malfunctioning	34	(65.4)	18	(34.6)	0	(0.0)	52	(100.0)
Sinusitis	30	(63.8)	16	(34.0)	1	(2.1)	47	(100.0)
Anomalies oral cavity	11	(57.9)	8	(42.1)	0	(0.0)	19	(100.0)
Disease of adenoid and tonsils	7	(53.8)	6	(46.2)	0	(0.0)	13	(100.0)
Vertigo	4	(44.4)	5	(55.6)	0	(0.0)	9	(100.0)
Osseous anomalies	4	(33.3)	8	(66.7)	0	(0.0)	12	(100.0)

## Discussion

In total 4536 patients were seen in PC+; 69.0% of them were referred back to the GP after PC+ consultation, whereas 29.1% were referred to secondary care. The outcome was unknown in 2.9% of patients. There was wide variation in the rate of referral to secondary care after PC+ consultation between specialties (8.6% (gynaecology) to 43.8% (orthopaedic surgery)), specialists (14.5 to 65.2%) and diagnosis groups (11.1 to 93.4%). These differences underline that, when participating in substitution initiatives like PC+, some specialties, specialists and diagnosis groups seem more appropriate than others. Differences between specialties might be explained by a variation in the need for additional in-hospital facilities and the possibility to equip PC+ consultation rooms with diagnostic devices. Differences between specialists from the same specialty may be caused by the individual way a specialist performs his or her occupation. A subspecialist who narrowed his scope down to a limited amount of diseases seems less equipped to work in a primary care setting than a specialist with a broader scope and interest in his field of specialization. Selection of specialists according to their profile might therefore reduce referrals to secondary care. Experience within PC+ may be another factor that influences referrals to secondary care; the more patients a specialist has seen in PC+, the better he is adapted to using fewer diagnostic tools, and as a consequence the fewer referrals to secondary care he is expected to make. Differences in referral patterns between diagnosis groups might be explained by the varying need for diagnosis specific in-hospital equipment. Whether referrals back to the GP reflect true substitution cannot be concluded based on this data, but will be subject to future reports about the PC+ intervention. Nonetheless, this information should be used to select appropriate specialties, specialists and patient groups for PC+. Combining those efforts might result in further declines of referral from PC+ to secondary care.

Few studies that explore the effectiveness of substitution initiatives have been published [7–14]. Most of them examined small-scale substitution initiatives with shared GP-specialist consultations in GP practices. Gruen et al. [10] reviewed a wide variety of studies evaluating the effectiveness of outreach clinic initiatives across the UK. This study showed ambiguous results regarding the effectiveness of outreach clinics in reducing referrals to secondary care. No clarity on specialty-specific referral patterns was provided. Vierhout et al. [11] explored the effects of shared consultations by GPs and orthopaedic surgeons. Within this study the orthopaedic surgeons held consultation hours in GP practices across the region of Maastricht in the Netherlands. The GP was able to attend the consultations in order to learn, share knowledge and deliberate with the specialist. Vierhout et al. found a 33.0% decline in referrals to secondary care within the intervention group compared to care as usual. Schulpen et al. [12, 13] found comparable results when exploring the effectiveness of joint consultations in GP practices for rheumatology patients, and described a positive learning effect for GPs after shared consultations. Van Hoof et al. [7, 8] presented comparable outcomes during a feasibility study of PC+ consultations in GP practices in 2013. Based on interviews with GPs and specialists it was expected that 32.0% of patients would remain in primary care after PC+ consultation. By contrast, exploring the current arrangement of PC+ in this study (specialist consultations in two out-hospital facilities) shows that 69.0% of all patients are sent back to the GP after consultation. This arrangement therefore seems most promising in achieving substitution of secondary care with primary care.

A report from NIVEL [5] indicated that care as usual referrals (i.e. without PC + -like initiatives) from the GP to secondary care vary between patient groups and are partly attributed to specialty-, GP- and patient group-specific characteristics. According to this report, in 2012 more female patients (303 per 1000) than male patients (220 per 1000) received a referral to secondary care, and older patients were likelier to end up in secondary care than younger patients. For both females (36%) and males (48%), most referrals were made for musculoskeletal complaints. Reflecting on the referral numbers in this study, it seems reasonable that the orthopaedic surgery and rheumatology departments refer most patients to secondary care. Also, gynaecology patients in this study were considerably younger than patients from other departments, which might partly explain the low number of referrals to secondary care. Further identification of specialty-, specialist- and patient group-specific factors that influence the chances of a referral to secondary care might be helpful in deciding which parts of secondary care are appropriate for substitution with primary care.

### Limitations

This study found differences in referrals after PC+ between several specialties, specialist and diagnosis groups. The data however, does not provide explanations for these differences that could be beneficial when improving substitution initiatives like PC+. Also, a small discrepancy between referral numbers based on the PC+ databases and true referral information from the Maastricht University Medical Centre was found, which could indicate that referral numbers from the PC+ databases do not correctly reflect actual patient flows. If considerable amounts of patients end up in secondary care shortly after a PC+ consultation, PC+ might, as a consequence, become an additional step in between primary and secondary care, generating double care loops instead of substitution. As was underlined by the head of the department of otorhinolaryngology, the fact that the department still treats roughly 8500 patients per year inside the hospital, as it did prior to the PC+ project, and an additional 1000 patients in PC+, could indicate that PC+ leads to a supply-driven demand. However, this could have also resulted from a priming effect of PC+ on patients from other regions, since waiting times to access specialist care have decreased after the foundation of PC+. This effect is not necessarily unbeneficial because GPs potentially refer their patients in an earlier stage to PC+ than they would to secondary care, which could prevent delays in diagnosis and treatment and, as a consequence result in higher quality of care. Analysis of patient flows on a national level, as well as comparative analyses of health outcomes will be conducted to address these questions. Also, since several specialties make use of a second consultation for a considerable number of their patients, and as equipment in PC+ consultation rooms is expanding with costly medical devices that do not reflect a primary care setting, a situation in which PC+ is hitting the target of fewer referrals to secondary care but missing the point of substitution of low complex hospital care might develop. Improving referral numbers from PC+ by transforming the PC+ facilities into small-scale hospitals is undesirable and might eventually lead to higher care costs. Finally, when generalizing this data, two main region specific factors should be considered that impact the feasibility to achieve substitution. First, in regions like Maastricht-Heuvelland, specialists receive a fixed salary regardless of the number of visits to the outpatient clinic of the hospital, which increases the willingness to adopt substitution initiatives like PC+ among specialists. This could translate into improved substitution compared to regions where a declining number of patients seen in secondary care does directly affect the specialists’ income. Therefore, varying specialist reimbursement agreements are important region specific factors that should be considered when generalizing this data. Second, specialists with a more generalist approach seem better equiped to work in initiatives like PC+, as opposed to super-specialists. Since the Maastricht University Medical Centre fulfils both the role of a highly specialized tertiary care provider and a regional hospital where non-super-specialized routine care is being delivered, improved substitution is expected since more generalist doctors could have taken part in the PC+ initiative. Substitution could be hampered when hospitals that merely deliver highly specialized care take part in these initiatives.

## Conclusion

The differences in referral numbers between specialties, specialists and diagnosis groups indicate that appropriate selection is important in optimising referrals from substitution initiatives like PC+ back to the GP. However, it is too early to draw firm conclusions regarding the amount of substitution that is taking place as part of achieving the Triple Aim dimensions with regard to cost reduction. Therefore, the prospective observational study that is currently ongoing will compare the effects of PC+ with other regions without the intervention in a comprehensive manner with the inclusion of financial data, patient satisfaction measures and the populations’ health. Nevertheless, these data indicate that selection of appropriate specialties, specialists and patient groups to participate in substitution initiatives like PC+ is vital in achieving lower referral numbers to secondary care.

## Abbreviations

DTC: Diagnosis-Treatment-Combination
GP: General Practitioner
ICD-10: International Statistical Classification of Diseases and Related Health Problems
ICPC: International Code Primary Care
OECD: Organization for Economic Cooperation and Development
PC+: Primary Care Plus
PFPS: Patello-Femoral Pain Syndrome
SC: Secondary Care
TIPP: Transmural Interactive Patient Platform
UK: United Kingdom
